# Air pollution in association with mental and self-rated health and the mediating effect of physical activity

**DOI:** 10.1186/s12940-022-00839-x

**Published:** 2022-03-07

**Authors:** Pauline Hautekiet, Nelly D. Saenen, Stefaan Demarest, Hans Keune, Ingrid Pelgrims, Johan Van der Heyden, Eva M. De Clercq, Tim S. Nawrot

**Affiliations:** 1 Risk and Health Impact Assessment, Sciensano, Juliette Wytsmanstraat 14, 1050 Brussels, Belgium; 2 Centre for Environmental Sciences, Hasselt University, 3500 Hasselt, Belgium; 3 Epidemiology and Public Health, Sciensano, Juliette Wytsmanstraat 14, 1050 Brussels, Belgium; 4 Centre of General Practice, University of Antwerp, Doornstraat 331, 2610 Antwerp, Belgium; 5 Nature and Society, Own-Capital Research Institute for Nature and Forest (EV-INBO), Vlaams Administratief Centrum Herman, Teirlinckgebouw, Havenlaan 88 bus 73, 1000 Brussels, Belgium; 6 Applied Mathematics, Computer Science and Statistics, Ghent University, Krijgslaan 281, S9, 9000 Gent, Belgium; 7 Department of Public Health and Primary Care, Environment and Health Unit, Leuven University, Herestraat 49, 3000 Leuven, Belgium

**Keywords:** Ambient air pollution, Depressive disorder, Self-rated health, Vitality, Belgium

## Abstract

**Background:**

Recent studies showed that air pollution might play a role in the etiology of mental disorders. In this study we evaluated the association between air pollution and mental and self-rated health and the possible mediating effect of physical activity in this association.

**Methods:**

In 2008, 2013 and 2018 the Belgian Health Interview Survey (BHIS) enrolled 16,455 participants who completed following mental health dimensions: psychological distress, suboptimal vitality, suicidal ideation, and depressive and generalized anxiety disorder and self-rated health. Annual exposure to nitrogen dioxide (NO_2_), particulate matter ≤ 2.5 µm (PM_2.5_) and black carbon (BC) were estimated at the participants’ residence by a high resolution spatiotemporal model. Multivariate logistic regressions were carried out taking into account a priori selected covariates.

**Results:**

Long-term exposure to PM_2.5_, BC and NO_2_ averaged 14.5, 1.4, and 21.8 µg/m^3^, respectively. An interquartile range (IQR) increment in PM_2.5_ exposure was associated with higher odds of suboptimal vitality (OR = 1.27; 95% CI: 1.13, 1.42), poor self-rated health (OR = 1.20; 95% CI: 1.09, 1.32) and depressive disorder (OR = 1.19; 95% CI: 1.00, 1.41). Secondly, an association was found between BC exposure and higher odds of poor self-rated health and depressive and generalized anxiety disorder and between NO_2_ exposure and higher odds of psychological distress, suboptimal vitality and poor self-rated health. No association was found between long-term ambient air pollution and suicidal ideation or severe psychological distress. The mediation analysis suggested that between 15.2% (PM_2.5_-generalized anxiety disorder) and 40.1% (NO_2_-poor self-rated health) of the association may be mediated by a difference in physical activity.

**Conclusions:**

Long-term exposure to PM_2.5_, BC or NO_2_ was adversely associated with multiple mental health dimensions and self-rated health and part of the association was mediated by physical activity. Our results suggest that policies aiming to reduce air pollution levels could also reduce the burden of mental health disorders in Belgium.

**Supplementary Information:**

The online version contains supplementary material available at 10.1186/s12940-022-00839-x.

## Background

Mental health has been defined by the WHO as “a state of well-being in which the individual realizes his or her own abilities, can cope with the normal stresses of life, can work productively and fruitfully, and is able to make a contribution to his or her community” [1]. World-wide, 14% of the global burden of all diseases can be attributed to mental, neurological, and substance use disorders [2]. Mental health is associated with multiple personal determinants, including gender [3], social support [4], physical activity [5] and socio-economic status [6]. Besides these well-known risk factors, environment is also important in this context [7] as increasing evidence suggests that higher levels of air pollution might affect mental health. Long-term exposure to nitrogen dioxide (NO_2_) and fine (≤ 2.5 µm (PM_2.5_)) and coarse (≤ 10 µm (PM_10_)) particulate matter has been associated with increased odds of depressive disorder [8–10]. Additionally, exposure to PM_2.5_ was associated with increased symptoms of anxiety disorder [11]. Finally, several studies showed a higher risk of suicidal ideation and suicide death among people exposed to higher long-term [12, 13] and short-term air pollution [14–16] concentrations.

Studies on this topic often include only one mental health outcome or one type of air pollutant. However, mental health is a broad concept and different air pollutants might induce different effects. For example, NO_2_ and black carbon (BC) are known as traffic-related air pollutants whereas PM_2.5_ is a complex multi-pollutant mixture of solid and liquid particles. As traffic only represents part of the PM exposure, the effects of PM might differ from the traffic-related pollutants [17, 18]. Therefore, in the first objective of this large cross-sectional study, we assessed self-rated health and multiple dimensions of mental health, i.e., psychological and severe psychological distress, suboptimal vitality, suicidal ideation, and depressive and generalized anxiety disorder in association with long-term exposure to PM_2.5_, BC and NO_2_.

Furthermore, research showed that higher concentrations of air pollution may be adversely associated with physical activity as air pollution could make a neighborhood environment less appealing for outdoor recreation [19]. In turn, being less active might negatively affect the mental health status [20, 21]. As shown by several reviews and meta-analyses, long-term exercise has anti-inflammatory effects [22], is protective against oxidative stress [23], promotes self-perception [24], and improves body image [25], which all positively affects mental health [20]. Also, during physical activity one might be exposed to higher air pollution concentrations, due to for example higher respiration rates [26]. This could diminish the positive effect of physical activity. However, research showed that the benefits of active travel outweighed the harm caused by air pollution, except for the most extreme concentrations [27]. As research on the topic of physical activity and air pollution is very limited, as a second objective, we evaluated the mediation effect of physical activity in the association between air pollution and mental and self-rated health. Our hypothesis is that exposure to higher air pollution concentrations is adversely associated with mental and self-rated health and that part of this association might be mediated by physical activity.

## Methods

### Study population

This cross-sectional study is executed on a subsample of the Belgian Health Interview Survey (BHIS) of 2008, 2013 and 2018. The sampling frame of the BHIS was the Belgian National Register and participants were selected based on a multistage stratified sampling design including a geographical stratification, a selection of municipalities within provinces, of households within municipalities, and of respondents within households [28]. The participation rate of the households in 2008, 2013 and 2018 was respectively 55.0%, 57.0%, and 57.5%.

In 2008, 2013 and 2018, in total 26,272 BHIS participants were eligible to complete the mental health modules (participants who participated through a proxy respondent (a person of confidence filled out the survey) or under 15 years old were not eligible). Our main analysis included 16,455 participants: as (1) data on air pollution and moving history of 581 participants was not available, (2) persons living less than one year on the current address were excluded (*n* = 2,984), and (3) participants who did not answer all mental and self-rated health questions and completed all information used as covariates in this study were excluded (*n* = 5,717 and 535, respectively), see flowchart in Supplementary Fig. 1.

Supplementary Table 1 shows the characteristics of the original 26,272 BHIS participants. Slightly more participants of Flanders and less of the Brussels Capital Region and more participants born in Belgium and with a higher educational level in the household were included. No other differences were found.

### Health interview survey

The BHIS is a comprehensive survey which aims to gain insight in the health status, health behavior and medical consumption of the Belgian population. The questions on mental health were based on international standardized and validated questionnaires [29]. Detailed information on each indicator score and its use is addressed in Supplementary Table 2.

According to the WHO, mental health includes the absence of mental illnesses but also the positive dimensions of mental health [1]. For the first part, the General Health Questionnaire (GHQ-12) aims at screening psychological wellbeing and detects possible disorders in general whereas the Symptom Checklist-90-Revised (SCL-90-R) focusses specifically on depressive and anxiety disorder. For the second part, the Short Form Health Survey (SF-36) contains 4 questions related to energy and vitality. More specifically, firstly, the GHQ-12 provides the prevalence of psychological and severe psychological distress in the population [30]. On the total GHQ-score cut-off points of 2 or more and 4 or more were used to identify respectively psychological and severe psychological distress. Secondly, four questions of the SF-36 indicate the participant’s vital energy level [31]. We used a cut-off point to identify those participants with an optimal vitality score, which is a score equal to or above the standard deviation above the mean, as used in previous studies [32, 33]. The standardized Cronbach’s alpha coefficients for the four questions related to vitality based on the SF-36 and for the GHQ-12 questionnaire were respectively 0.79 and 0.89.

Over the BHIS data collection years, there have been changes in the selection of instruments for assessing depressive and generalized anxiety disorder. Therefore, the indicators based on these instruments were evaluated in a subset containing only the participants of 2008 and 2013 (*n* = 10,153). In these years, the SCL-90-R questionnaire was used. A score higher than 1 identified those participants who suffer from depressive or generalized anxiety disorder [34]. The standardized Cronbach’s alpha coefficients for the SCL-90-R scales for generalized anxiety disorder and depressive disorder were respectively 0.90 and 0.92.

Other indicators in the BHIS were based on single questions. Firstly, a dichotomous question on suicidal ideation in the past 12 months was used: “Have you ever seriously thought of ending your life?”; “If yes, did you have such thoughts in the past 12 months?”. Secondly, the question “How is your health in general? Is it very good, good, fair, poor, or very poor?” was used to indicate self-rated health [35]. The answer categories were dichotomized into good self-rated health (very good to good) and poor self-rated health (fair, poor, and very poor). Finally, the BHIS also included personal, socio-economic and lifestyle information.

### Air pollution assessment

Residential addresses of the participants were geocoded. Daily residential exposure (µg/m^3^) to PM_2.5_, BC and NO_2_ at the participants’ residence was modelled at high resolution using a spatiotemporal interpolation model [36]. This model included air pollution data from the Belgian fixed monitoring stations and CORINE Land Cover (CLC) information obtained by satellites in combination with a dispersion model including point and line sources [37–39]. The overall model performance was evaluated by leave-one-out cross-validation and was based on 34 monitoring points for PM_2.5_, 44 for NO_2_ and 14 for BC. The temporal and spatial variability of the model was explained by 80% for PM_2.5_ [39], 78% for NO_2_ [39] and 74% for BC [38]. Furthermore, during a previous study, we validated the modelled exposure by measuring the internal carbon as urinary carbon particles, showing that the internal carbon exposure is correlated with the chronic residential carbon exposure (*partial r* = 0.17, CI: 0.05 to 0.28) [40]. We used the daily modelled exposure values obtained by this model to calculate the residential exposure during the year of participation. Because the high resolution model was not available for the year 2008, the data of 2009–10 was used as a representative for the spatial contrast of the earlier period. Evidence showed that the spatial distribution of particulate air pollution is stable over a decade and that existing land use regression models are good predictors of historical spatial contrasts [41–44].

### Covariates and mediators

Covariates included in the models were age (continuous), gender (male; female), year of participation (2008; 2013; 2018), region (Flanders; Brussels Capital Region; Wallonia), highest educational level of the household (up to lower secondary; higher secondary; college or university), country of birth (Belgian; EU; non-EU), household composition (single; one parent with child(ren); couple without child(ren); couple with child(ren); other) and smoking status (current smoker; current non-smoker). To capture the non-linear effect of age, we included a quadratic term when the result of the analysis showed that both the linear and quadratic term were significant.

Physical activity was based on the question ‘What describes best your leisure time activities during the last year?’ Possible answer categories were: (1) Hard training and competitive sport more than once a week; (2) Jogging and other recreational sports or gardening, 4 h or more per week; (3) Jogging and other recreational sports or gardening, less than 4 h per week; (4) Walking, bicycling or other light activities 4 h or more a week; (5) Walking, bicycling or other light activities less than 4 h a week; and (6) Reading, watching TV or other sedentary activities. Category 1 to 5 were combined to present active lifestyle whereas category 6 represents sedentary lifestyle. A directed acyclic graph including all covariates and the mediator is presented in Supplementary Fig. 2.

### Statistical analysis

We performed single exposures multivariate logistic regressions taking into account the previously mentioned a priori selected covariates. Furthermore, we accounted for the complex survey design by including clustering and stratification in the model. The variance inflation factor (VIF) was calculated to assess possible multi-collinearity between the explanatory variables. A VIF value of 5 was used as threshold. All results are presented as the odds ratio (95% CI) of having a mental health condition or disorder or a poor-self rated health for an IQR increment in air pollution exposure. Chi-squared tests (categorical data) and t-tests (continuous data) were used to evaluate differences in characteristics between participants with and without psychological distress.

In a secondary analysis, we performed a mediation analysis to determine whether physical activity was a potential mediator of the association between air pollution and mental and self-rated health. This analysis was performed on a subset of 14,899 participants. The direct effect (DE), indirect effect (IE), total effect (TE) and proportion mediated were estimated using the CAUSALMED procedure in SAS, in a counterfactual framework, based on the SAS macro by Valeri and VanderWeele [45]. PROC CAUSALMED estimates effects after maximum likelihood estimation and uses the delta method to estimate the standard errors. An interaction term between exposure and mediator was included in the model. When assumptions of the mediation analysis hold, the DE represents the effect of air pollution exposure on mental or self-rated health independent from physical activity and the IE represents the estimated effect of air pollution exposure operating through physical activity [45]. The covariates for which we adjusted in the mediation models were as same as in the main models. Statistical analyses were performed using SAS software (version 9.4; SAS Institute Inc., Cary, NC, USA). GIS analyses to calculate green space were carried out using ArcGIS 10 software.

### Sensitivity analysis

In a first sensitivity analysis, models were additionally adjusted for urbanization level (big cities/dense agglomerations; suburban areas; urbanized municipalities; rural areas), physical activity (active lifestyle; sedentary lifestyle), and green space in a buffer of 1000 m around the participants’ residence (continuous), separately. Physical activity [46] is known to be associated with mental health whereas urbanization level [47] and green space [48] are known to be associated with air pollution. Green space was calculated with Corine land Cover data of 2012 (European Environment Agency) and included the classes of forest and semi natural areas, wetlands, water bodies and artificial, non-agricultural vegetated areas.

Secondly, the SF-36 and SCL-90-R questionnaires were used with one cut-off point. In a second sensitivity analysis, we evaluated the effect of air pollution on the continuous scale of the SF-36. Linear regressions were performed adjusted for the same covariates as the main analyses and taking into account clustering and stratification. For the SCL-90-R no normal distributed residuals were found. Therefore, we divided the score in tertiles and evaluated those three groups, using the same model as for the main analyses. Class 0 indicates the lowest scores, class 2 indicates the highest scores.

Finally, in a third sensitivity analysis, to evaluate effect modification by age, gender and socio-economic status (highest educational level of the household), interaction terms for these covariates with the exposure were added separately to the models. Stratification was done when the interaction term showed a significance ≤ 0.10. As age was used as a continuous parameter, four groups were made for stratification (15–29; 30–44; 45–59; + 60).

## Results

### Population characteristics

The characteristics of the 16,455 participants included in this study are presented in Table 1. Out of all participants, 47.5% were men and the average age ± SD was 50.3 ± 18.9 years. The table also shows large differences in characteristics between participants with and without psychological distress.

**Table 1:** Characteristics of all BHIS participants (*n* = 16,455) and categorized as with (*n* = 4,932) and without (*n* = 11,523) psychological distress

Characteristics	All BHIS participants *n* (%) or mean ± *SD*	With psychological distress *n* (%) or mean ± *SD*	Without psychological distress *n* (%) or mean ± *SD*	*p*-value
Male	7,813 (47.5%)	1,959 (39.7%)	5,854 (50.8%)	< 0.0001
Age, years	50.3 ± 18.9	48.9 ± 18.9	50.9 ± 18.9	< 0.0001
Year				< 0.0001
2008	5,479 (33.3%)	1,498 (30.4%)	3,981 (34.6%)	
2013	4,674 (28.4%)	1,435 (29.1%)	3,239 (28.1%)	
2018	6,302 (38.3%)	1,999 (40.5%)	4,303 (37.3%)	
Region				< 0.0001
Flanders	6,946 (42.2%)	1,856 (37.6%)	5,090 (44.2%)	
Brussels Capital Region	3,538 (21.5%)	1,246 (25.3%)	2,292 (19.9%)	
Wallonia	5,971 (36.3%)	1,830 (37.1%)	4,141 (35.9%)	
Highest educational level in the household				0.014
Up to lower secondary school	3,543 (21.5%)	1,132 (23.0%)	2,411 (20.9%)	
Higher secondary school	5,149 (31.3%)	1,526 (30.9%)	3,623 (31.4%)	
College or university	7,763 (47.2%)	2,274 (46.1%)	5,489 (47.6%)	
Household composition				< 0.0001
Single	3,575 (21.7%)	1,238 (25.1%)	2,337 (20.3%)	
One parent with child(ren)	1,349 (8.2%)	565 (11.5%)	784 (6.8%)	
Couple without child(ren)	4,357 (26.5%)	1,085 (22.0%)	3,272 (28.4%)	
Couple with child(ren)	6,080 (37.0%)	1,708 (34.6%)	4,372 (37.9%)	
Other	1,094 (6.7%)	336 (6.8%)	758 (6.6%)	
Country of birth				0.0001
Belgium	13,841 (84.1%)	4,122 (83.6%)	9,719 (84.3%)	
EU	1,374 (8.4%)	378 (7.7%)	996 (8.6%)	
Non-EU	1,240 (7.5%)	432 (8.8%)	808 (7.0%)	
Smoking status				< 0.0001
Current smoker	3,455 (21.0%)	1,256 (25.5%)	2,199 (19.1%)	
Current non-smoker	13,000 (79.0%)	3,676 (74.5%)	9,324 (80.9%)	
Physical activity ^a^				< 0.0001
Active lifestyle	10,413 (69.9%)	2,761 (62.1%)	7,652 (73.2%)	
Sedentary lifestyle	4,486 (30.1%)	1,686 (37.9%)	2,800 (26.8%)	
Urbanization ^b^				< 0.0001
Big cities / dense agglomerations	7,178 (44.0%)	2,331 (47.6%)	4,847 (42.4%)	
Suburban areas	2,326 (14.2%)	642 (13.1%)	1,684 (14.7%)	
Urbanized municipalities	4,083 (25.0%)	1,149 (23.5%)	2,934 (25.7%)	
Rural areas	2,743 (16.8%)	776 (15.8%)	1,967 (17.2%)	
Green space 1000 m buffer	8.1% ± 11.9	7.8% ± 11.7	8.2% ± 12.0	0.118

^a^ data available for 14,899 participants in total and 4,447 and 10,452 with and without psychological distress respectively

^b^ data available for 16,330 participants in total and 4,898 and 11,432 with and without psychological distress respectively

Population data from the survey years indicate that the average age of the Belgian population over 15 years old was 47.6 (19.5), 48.7% were men and the distribution over Flanders, Brussels Capital Region and Wallonia was respectively 58.1%, 10.0%, 31.9%. The distribution of our sample according to age and gender thus largely corresponds to the Belgian 15 + population figures. The difference in the regional distribution is due to the oversampling of the Brussels Capital Region in the BHIS. Table 2 shows the prevalence of each of the mental health dimensions and self-rated health.

**Table 2:** Prevalence of the different mental health dimensions and self-rated health in the BHIS (*n* = 16,455)

Mental health dimensions	Prevalence (%)
Psychological distress	30.0%
Severe psychological distress	16.6%
Suicidal ideation	4.4%
Suboptimal vitality	85.6%
Depressive disorder ^a^	12.7%
Generalized anxiety disorder ^a^	7.9%
Poor self-rated health	23.2%

^a^ subset (*n* = 10,153)

### Air pollutant exposure levels

The air pollutant exposure levels are presented in Table 3. The average annual PM_2.5_, BC and NO_2_ concentrations (IQR) were respectively 14.5 (3.8) µg/m^3^, 1.4 (0.6) µg/m^3^ and 21.8 (10.8) µg/m^3^. The air pollutants were highly correlated. The Spearman correlation coefficients between PM_2.5_ and BC, PM_2.5_ and NO_2_ and BC and NO_2_ were respectively 0.86 (*p* < 0.0001), 0.74 (*p* < 0.0001) and 0.85 (*p* < 0.0001).

**Table 3:** Annual ambient residential exposure characteristics (µg/m^3^)

	**Mean**	**25**^**th**^** percentile**	**50**^**th**^** percentile**	**75**^**th**^** percentile**	**IQR**
PM_2.5_	14.5	12.8	14.4	16.5	3.8
BC	1.4	1.0	1.2	1.6	0.6
NO_2_	21.8	16.2	20.3	27.0	10.8

### Air pollution and mental and self-rated health

**Fig. 1:**
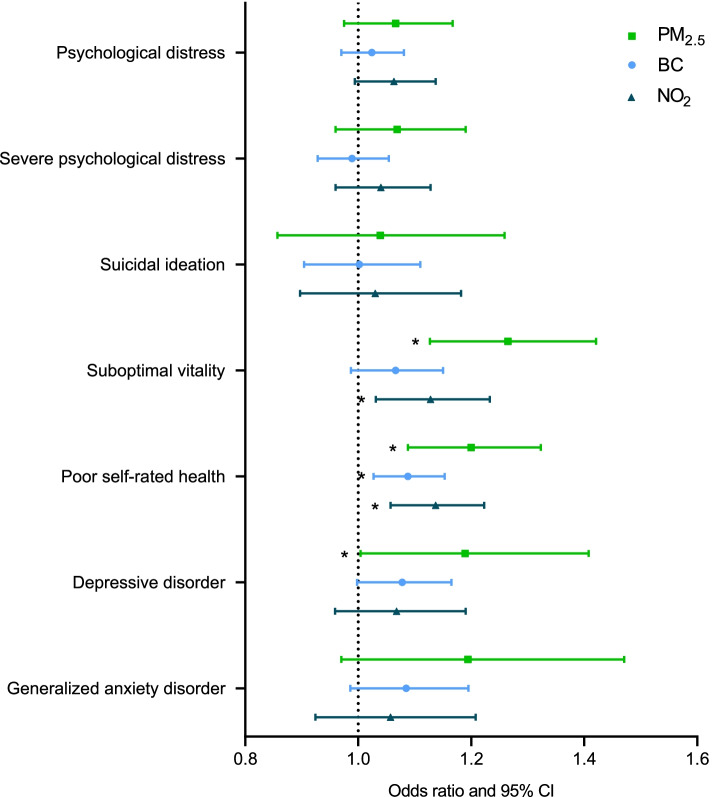
Odds ratios (with 95% CI) of all mental health dimensions and self-rated health for an IQR increment in annual PM_2.5_, NO_2_ or BC concentration. Estimates were adjusted for age, gender, year of participation, region, country of birth, household composition, smoking status, highest educational level of the household and the quadratic term of age when both the linear and quadratic term were significant. *p ≤ 0.05. IQRs for PM_2.5_, BC, and NO_2_ were respectively 3.8 µg/m^3^, 0.6 µg/m^3^ and 10.8 µg/m^3^

All results are presented in Fig. 1 and estimates are available in Supplementary Table 3. Firstly, our results showed significant associations between long-term PM_2.5_ exposure and various mental health outcomes and self-rated health. Each 3.8 µg/m^3^ increment in long-term PM_2.5_ (IQR contrast) was associated with increased odds of suboptimal vitality (OR = 1.27; 95% CI: 1.13, 1.42), poor self-rated health (OR = 1.20; 95% CI: 1.09, 1.32) and depressive disorder (OR = 1.19; 95% CI: 1.00, 1.41). Secondly, an IQR increment in BC (0.6 µg/m^3^) was associated with increased odds of poor self-rated health (OR = 1.09; 95% CI: 1.03, 1.15), depressive disorder (OR = 1.08; 95% CI: 1.00, 1.17) and generalized anxiety disorder (OR = 1.09; 95% CI: 0.99, 1.20). Finally, we found for an IQR increment in NO_2_ exposure (10.8 µg/m^3^) increases in the odds of psychological distress (OR = 1.06; 95% CI: 0.99,1.14), suboptimal vitality (OR = 1.13; 95% CI: 1.03, 1.23) and poor self-rated health (OR = 1.14 95% CI; 1.06, 1.22). No association was found between long-term ambient air pollution and suicidal ideation or severe psychological distress. The average VIF value was 1.43.

### Mediation analysis

**Fig. 2:**
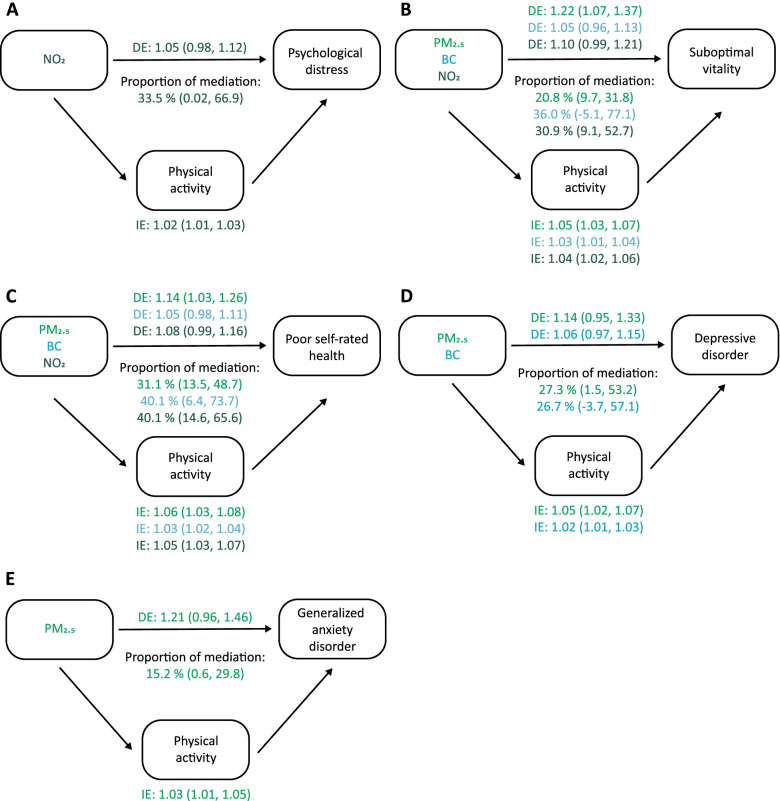
Estimated effect of air pollution exposure on mental health and self-rated health mediated through physical activity. The figure displays air pollution (PM_2.5_, BC or NO_2_) as treatment, physical activity as mediator and mental or self-rated health as outcome (**A** psychological distress, **B** suboptimal vitality, **C** poor self-rated health, **D** depressive disorder, **E** generalized anxiety disorder). The figure shows the odds ratios (95% CI) of the indirect effect (IE) and the direct effect (DE), and the proportion of mediation. The mediation model was adjusted for age, gender, year of participation, region, country of birth, household composition, smoking status and highest educational level of the household

A mediation analysis was conducted to evaluate the potential indirect effect of physical activity in the association between air pollution and the indicators of mental health and self-rated health. For all studied ambient pollutants, higher levels were associated with higher odds of having a sedentary lifestyle. Secondly, for all mental health outcomes and self-rated health, the results showed that the odds of having a mental health condition or disorder or having a poor self-rated health was higher for those having a sedentary lifestyle (Suppl. Table 4). The results of the mediation analysis suggested that between 15.2% and 40.1% (Fig. 2) of the association between air pollution (mostly PM_2.5_) and mental or self-rated health was mediated by physical activity, with exception of severe psychological distress and suicidal ideation (Suppl. Table 5). The strongest mediation effect was found for the association between BC and NO_2_ and poor self-rated health: 40.1% (95% CI: 6.4%, 73.7%) and 40.1% (95% CI: 14.6%, 65.5%), respectively.

### Sensitivity analysis

In a sensitivity analysis, additional adjustment for physical activity, urbanization level, or green space did not change the effect direction observed in our main analyses (Suppl. Table 3). However, additional adjustment for physical activity resulted in loss of significance in multiple associations and additional adjustment for urbanization showed higher odds for generalized anxiety disorder when exposed to an IQR increase in PM_2.5_ (OR = 1.33, 95% CI: 1.03, 1.71).

Secondly, when evaluating the SF-36 score continuously, an IQR increase in PM_2.5_, BC and NO_2_ was associated with respectively a -2.40 (95% CI: -3.13, -1.67, *p* < 0.0001), -0.83 (95% CI: -1.31, -0.36, *p* = 0.0005), and -1.48 (95% CI: -2.04, -0.94, *p* < 0.0001) lower vitality score. This is in line with the associations based on the dichotomous score. Also, when evaluating depressive and generalized anxiety disorder in tertiles, an IQR increment in PM_2.5_ was associated with higher odds of being in class 1 (OR = 1.15, 95% CI: 1.01, 1.30) and class 2 (OR = 1.15, 95% CI: 1.00, 1.32) compared with class 0 for depressive disorder. For the same exposure, a trend indicates higher odds of being in class 2 (OR = 1.37, 95% CI: 0.95, 1.95) compared with class 0 for anxiety disorder (Suppl. Table 6).

Finally, results of effect modification can be found in Supplementary tables 7-9. Firstly, the association between poor self-rated health and both PM_2.5_ and BC and between severe psychological distress and PM_2.5_ was mainly driven by the elderly (+ 60 years). Secondly, the association between PM_2.5_ and depressive and generalized anxiety disorder was stronger for women compared with men whereas the opposite was found for the association between psychological distress and PM_2.5_ and NO_2_. Finally, no clear indication for effect modification by socio-economic status could be demonstrated.

## Discussion

In this study, we demonstrated that long-term ambient residential air pollution exposure was associated with different dimensions of mental health and self-rated health in the Belgian population. Furthermore, our results indicated that part of the association between air pollution and mental or self-rated health was mediated by physical activity and that the association between mental health outcomes and air pollution was stronger in elderly and in women. All discussed literature for each mental health outcome is combined in Supplementary Table 10.

### Air pollution and mental health

Our first results showed higher odds of psychological distress for a higher NO_2_ exposure. Only a few studies previously evaluated this association. In a fully-adjusted model, exposure to higher annual PM_2.5_ concentrations was associated with a higher level of psychological distress (β = 0.185; 95% CI: 0.079, 0.290), but for NO_2_, only an effect was found in the unadjusted analysis [49]. In another study in an adult population, for each IQR increment in long-term exposure to PM_2.5_ (0.82 µg/m^3^) and NO_2_ (7.85 µg/m^3^), the odds ratio of severe psychological distress was respectively 1.08 (95% CI: 1.06, 1.11) and 1.08 (95% CI: 1.05, 1.11) [50]. The different outcomes between our study and the two previously mentioned studies cannot be explained by the pollutant concentrations as these were similar as for our study. Also, these studies used the Kessel Psychological Distress Scale while we used the GHQ-12 questionnaire, but research showed that both questionnaires were positively associated [51].

Secondly, we found no significant association between long-term air pollution and suicidal ideation. Until now 11 studies assessed suicide in association with short-term air pollution [14, 15, 52], two studies with long-term air pollution [12, 13] and three studies included different time periods [53, 54]. In a study in Belgium, during summer, people were more likely to commit suicide when PM_10_ and ozone concentrations were high [14]. However, Casas et al. [14] showed acute effects while in this study the focus was long-term exposure to air pollution. In a study in South-Korea, where participants were followed up from 2002 until 2013 and were 564 (0.2%) participants died from suicide in this period, the risk of suicide was higher in the highest quartile for long-term PM_10_ (Hazard ratio = 4.03; 95% CI: 2.97, 5.47) and in the third quartile for annual NO_2_ (Hazard ratio = 1.52; 95% CI: 1.17, 1.96) compared with the lowest level of the air pollutants [12]. In another Korean study, yearly average exposure to the highest quartile of NO_2_ was associated with increased odds of suicidal ideation for men and women (OR = 1.32; 95% CI: 1.18, 1.48 and OR = 1.40; 95% CI: 1.29, 1.53 respectively) compared with the reference quartile. Similar results were found for PM_10_ [13]. One of the reasons why we did not find an association might be the low prevalence of suicidal ideation in our study population (4.4%) while in the study of Shin et al. (2018) this was 8.8% [13].

Thirdly, our results showed higher odds of suboptimal vitality with increasing PM_2.5_, BC and NO_2_ exposure. Similarly, Yamazaki and colleagues (2006) showed a significant linear association between one month exposure to ambient photochemical oxidants (O_x_), and a lower vitality score, according to the four questions in the SF-36 questionnaire on vitality [55]. However, the association with suspended particulate matter was only found for the crude analysis and no association was found for NO_x_.

Fourthly, we observed a positive association between the air pollutants and depressive disorder. This is in line with the conclusions of several systematic reviews [7, 56–58]. Additionally, in a subpopulation of 958 adults living in Barcelona, long-term NO_2_ and PM_2.5_ exposure was positively associated with depressive disorder, i.e., each 5 µg/m^3^ increment in PM_2.5_ was associated with increased odds of depressive disorder of 4.38 (95% CI: 1.70, 11.30) [10]. Also, in a 3-year follow-up study on elderly in Korea, an IQR increment in 3-day moving average of PM_10_ was associated with a 17% (95% CI: 4.9%, 30.5%) increment in the depression score [59]. In our study, the association between PM_2.5_ and depressive disorder was mainly driven by the women. Both previously mentioned studies did not evaluate effect modification by gender but their study populations included a high percentage of women (63.9% and 74% respectively).

Finally, we found that higher exposure to PM_2.5_ and BC was associated with higher odds of generalized anxiety disorder but this association was less clear for NO_2_. Previous literature is inconclusive. Two studies found significant associations between PM_2.5_ and anxiety disorder [8, 11], whereas Vert et al. [10] found no association of PM_2.5_, PM_10_ or NO_2_ exposure with anxiety disorder [10]. The use of different questionnaires might explain the different results. Also, differences might be explained by the study design as Vert et al. (2017), Pun et al. (2017) and Power et al. (2015) used respectively a cross-sectional, a longitudinal and a cohort study design. Furthermore, the latter included only women, which as mentioned previously, tend to have more mental health issues compared with men [60].

### Air pollution and self-rated health

All three air pollutants were positively associated with poor self-rated health. Nakao and colleagues (2019) found comparable results, i.e., one month exposure to higher NO_2_ concentrations and suspended particulate matter in Japan was significantly associated with a decreased overall health score. However, the association with NO_2_ was only present in one of the two explored cities and no association was found with PM_2.5_ [61]. Also, their participants ranged between 40 and 79 years old. This is consistent with our findings as our sensitivity analysis showed that the association between PM_2.5_ and BC and self-rated health was mainly driven by the elderly.

### Biological mechanisms

How air pollution might affect mental or self-rated health is still not fully understood. Animal studies have shown that particles can be transferred to the brain. For example, after 12 days of exposure to manganese oxide ultra-fine particles, the concentrations increased 3.5-fold in the olfactory bulb and to a lesser extent in other brain regions of rats [62]. Furthermore, a study in Mexico City showed elevated levels of inflammatory markers in the brain of children when exposed to higher concentrations of air pollution [63, 64]. The translocation of the particles to the brain can be explained by two hypotheses. They can either access the brain through the blood–brain barrier after they deposit in the lungs and translocate to the blood circulation or they can travel via the olfactory nerve after they deposit on the nasal olfactory mucosa [65, 66]. Via the olfactory nerve, odor information is sent to the olfactory bulb and the brain. The amygdala and hippocampus, which process odor information, are also part of the limbic system and thus responsible for emotion regulation [67, 68]. Through this connection, it is believed that odors can regulate mood and behavior [67]. Consequently, air pollution particles might affect the olfactory pathway and also modulate emotion regulation. However, research is warranted to support this theory.

### Mediation analysis

The results of our sensitivity analysis showed that after adjusting for physical activity, the significant associations between air pollution and mental or self-rated health were lost. This indicates that physical activity is a strong moderator in our study setup and could imply that the benefits of physical activity on mental health outweigh the harm caused by air pollution [27, 69]. Further investigation indicated that both the exposures and outcomes were strongly associated with physical activity. A higher exposure to air pollution was associated with a less active lifestyle, which in turn was associated with a poor mental health outcome. The results of our mediation analysis showed that a considerable proportion of the association between air pollution and mental and self-rated health was mediated by physical activity. Similar results were found by Wang et al. (2019) where the mediation of physical activity was evaluated in the association between PM_2.5_ and depression [9]. Another study showed no direct association between air pollution and general mental health but it was indirectly associated through annoyance, restorative quality, and physical activity, working in serial [70].

Importantly, caution is advised when interpreting these results. Firstly, the association between physical activity and mental health has been evaluated in multiple studies [20, 21] but it is not straight forward as it can go in both directions. A sedentary lifestyle might induce mental health disorders, whereas people with mental health disorders might be less willing to engage in physical activity. As mediation analysis assumes causality [45] and most, but still weak, evidence suggests the first hypothesis [71, 72], we assumed that physical activity affects mental health. Still, further research is warranted to prove causality of this association.

Secondly, only few studies evaluated the association between air pollution and physical activity [73–76] and the pathways are still unclear. Three hypothesis for this association are proposed in the reviews of Tainio et al. [19] and An et al. [77]. Firstly, media alerts and health professionals informing the public about daily changes in air pollution levels might change day-to-day decisions on physical activity [78]. Secondly, air pollution adversely affects respiratory and cardiovascular health [79, 80] which may result in a reduced exercise capacity and performance [81, 82]. Finally, visible air pollutants like smog might discourage people from engaging in outdoor physical activity [74]. Important to notice is that most of the limited evidence is based on the U.S. and China where air pollution concentrations may be higher compared with Belgium. Further research on this matter in Western Europe is warranted.

### Strengths and limitations

The strength of this study is the use of a large dataset and while other studies often only assess one mental health outcome, we were able to evaluate multiple mental health dimensions and self-rated health, based on validated tools. Also, we assessed three different air pollutants that were estimated with a high-resolution spatiotemporal model.

We also acknowledge some limitations of this study. Because the high resolution model was not available for the year 2008, the data of 2009–10 was used as a representative for the spatial contrast of the earlier period. Evidence showed that the spatial distribution of particulate air pollution is stable over a decade and that existing land use regression models are good predictors of historical spatial contrasts [41–44]. Furthermore, as air pollution is measured at the participants’ residence, risks might be underestimated due to exposure misclassification depending on their mobility or behavior pattern [83]. However, previously we have found that urinary particle load reflected the residential air pollution exposure [40]. Secondly, we did not adjust for noise, which can also affect mental health [84]. Data on noise was not available for this project and furthermore, this is measured differently in the different regions of Belgium. Finally, a limitation of this research is that the mediation model assumes causality, i.e. that physical activity precedes mental health. However, as mentioned in the discussion, the association between physical activity and mental health might be bidirectional. Due to the cross-sectional study design, it is not possible to identify the direction of the effect but only to identify associations.

## Conclusion

Our data support the association between long-term exposure to PM_2.5_, BC and NO_2_ and a poor mental and self-rated health status. Furthermore, we showed that part of the association can be explained by physical activity. Our results suggest that both lifestyle factors and environment exposures should be taken into account to improve mental health. Due to some inconsistency in the results and the limitations of our study design, future studies are warranted to confirm our results.

## Supplementary Information

**Additional file 1: Supplementary table 1.** Characteristics of the study population (*n* = 16,455) and the original BHIS population, eligible to complete the mental health questionnaires (*n* = 26,272). **Supplementary table 2.** The mental health indicators with their scores and uses. **Supplementary table 3.** Results of the main and the sensitivity analyses. **Supplementary table 4.** The results of physical activity associated with mental and self-rated health and air pollution. **Supplementary table 5.** The effects of air pollution exposure on mental and self-rated health mediated through physical activity. **Supplementary table 6.** Association between air pollution and depressive and generalized anxiety disorder where the outcomes are classified as tertiles. **Supplementary table 7.** Results of the effect modification of age. **Supplementary table 8.** Results of the effect modification of sex. **Supplementary table 9.** Results of the effect modification of socio-economic status (SES). **Supplementary table 10.** Overview of all literature presented in the discussion. **Supplementary figure 1.** Exclusion criteria. **Supplementary figure 2.** Directed acyclic graph (DAG) for the association between air pollution and mental and self-rated health. Red: Ancestor of exposure and outcome; Green line: causal path; Purple line: biasing path.

## Data Availability

The dataset used for this study is available through a request to the Health Committee of the Data Protection Authority.

## Abbreviations

BC: Black Carbon
CI: Confidence Intervals
CLC: Corine Land Cover
GHQ-12: General Health Questionnaire
IQR: Interquartile Range
NO_2_: Nitrogen dioxide
OR: Odds Ratio; PM_2.__5_: Particulate matter ≤ 2.5 µm
SF-36: Short Form Health Survey
SCL-90-R: Symptom Checklist 90 Revised
