# Supplementary material for: Air pollution in association with mental and self-rated health and the mediating effect of physical activity
Source: Environ Health. 2022 Mar 7;21:29. doi: 10.1186/s12940-022-00839-x (PMC8903639; doi:10.1186/s12940-022-00839-x)
Supplement: Supplementary file 1 — Additional file 1: Supplementary table 1. Characteristics of the study population (n = 16,455) and the original BHIS population, eligible to complete the mental health questionnaires (n = 26,272). Supplementary table 2. The mental health indicators with their scores and uses. Supplementary table 3. Results of the main and the sensitivity analyses. Supplementary table 4. The results of physical activity associated with mental and self-rated health and air pollution. Supplementary table 5. The effects of air pollution exposure on mental and self-rated health mediated through physical activity. Supplementary table 6. Association between air pollution and depressive and generalized anxiety disorder where the outcomes are classified as tertiles. Supplementary table 7. Results of the effect modification of age. Supplementary table 8. Results of the effect modification of sex. Supplementary table 9. Results of the effect modification of socio-economic status (SES). Supplementary table 10. Overview of all literature presented in the discussion. Supplementary figure 1. Exclusion criteria. Supplementary figure 2. Directed acyclic graph (DAG) for the association between air pollution and mental and self-rated health. Red: Ancestor of exposure and outcome; Green line: causal path; Purple line: biasing path. [file 12940_2022_839_MOESM1_ESM.docx]

**Air pollution in association with mental and self-rated health and the mediating effect of physical activity**

Pauline Hautekiet^1,2^, Nelly D. Saenen^1,2^, Stefaan Demarest^3^, Hans Keune^4,5^, Ingrid Pelgrims^1,3,6^, Johan Van der Heyden^3^, Eva M. De Clercq^1^, Tim S. Nawrot^2,7^

^1^ Risk and health impact assessment, Sciensano, Juliette Wytsmanstraat 14, BE-1050, Brussels, Belgium

^2^ Centre for Environmental Sciences, Hasselt University, BE-3500, Hasselt, Belgium

^3^ Epidemiology and public health, Sciensano, Juliette Wytsmanstraat 14, BE-1050, Brussels, Belgium

^4^ Primary and Interdisciplinary Care, University of Antwerp, Prinsstraat 13, BE-2000, Antwerp, Belgium

^5^ Nature and society, Own-Capital Research Institute for Nature and Forest (EV-INBO), Vlaams Administratief Centrum Herman, Teirlinckgebouw, Havenlaan 88 bus 73, BE-1000, Brussels, Belgium

^6^ Applied Mathematics, Computer Science and Statistics, Ghent University, Krijgslaan 281, S9, BE-9000, Gent, Belgium

^7^ Centre for Environment and Health, Leuven University, BE-3000, Leuven, Belgium

Corresponding author: Tim S. Nawrot, tim.nawrot@uhasselt.be, Centre for Environmental Sciences, Hasselt University, BE-3500, Hasselt, Belgium

**Supplementary table 1: Characteristics of the study population (n = 16,455) and the original BHIS population, eligible to complete the mental health questionnaires (n = 26,272).**

| **Characteristics** | **Study population**  **n (%) or mean ± SD** | **n (original BHIS**  **population)** | **Original BHIS population**  **n (%) or mean ± SD** |
| --- | --- | --- | --- |
| **Male** | 7,813 (47.5%) | 26,272 | 12,312 (46.9%) |
| **Age, years** | 50.3 ± 18.9 | 26,272 | 50.3 ± 19.8 |
| **Year** |  | 26,272 |  |
| **2008** | 5,479 (33.3%) |  | 8,623 (32.8%) |
| **2013** | 4,674 (28.4%) |  | 7,896 (30.1%) |
| **2018** | 6,302 (38.3%) |  | 9,753 (37.1%) |
| **Region** |  | 26,272 |  |
| **Flanders** | 6,946 (42.2%) |  | 9,644 (36.7%) |
| **Brussels Capital Region** | 3,538 (21.5%) |  | 6,864 (26.1%) |
| **Wallonia** | 5,971 (36.3%) |  | 9,764 (37.2%) |
| **Highest educational level in the household** |  | 25,757 |  |
| **Up to lower secondary school** | 3,543 (21.5%) |  | 6,628 (25.7%) |
| **Higher secondary school** | 5,149 (31.3%) |  | 8,054 (31.3%) |
| **College or university** | 7,763 (47.2%) |  | 11,075 (43.0%) |
| **Household composition** |  | 26,272 |  |
| **Single** | 3,575 (21.7%) |  | 6,220 (23.7%) |
| **One parent with child(ren)** | 1,349 (8.2%) |  | 2,323 (8.8%) |
| **Couple without child(ren)** | 4,357 (26.5%) |  | 6,852 (26.1%) |
| **Couple with child(ren)** | 6,080 (37.0%) |  | 8,899 (33.9%) |
| **Other** | 1,094 (6.7%) |  | 1,978 (7.5%) |
| **Country of birth** |  | 25,636 |  |
| **Belgium** | 13,841 (84.1%) |  | 20,373 (79.5%) |
| **EU** | 1,374 (8.4%) |  | 2,492 (9.7%) |
| **Non-EU** | 1,240 (7.5%) |  | 2,771 (10.8%) |
| **Smoking status** |  | 21,635 |  |
| **Current smoker** | 3,455 (21.0%) |  | 4,827 (22.3%) |
| **Current non-smoker** | 13,000 (79.0%) |  | 16,808 (77.7%) |
| **Physical activity ^a^** |  | 19,555 |  |
| **Active lifestyle** | 10,413 (69.9%) |  | 13,463 (68.9%) |
| **Sedentary lifestyle** | 4,486 (30.1%) |  | 6,092 (31.2%) |
| **Urbanization ^b^** |  | 26,077 |  |
| **Big cities / dense agglomerations** | 7,178 (44.0%) |  | 12,259 (47.0%) |
| **Suburban areas** | 2,326 (14.2%) |  | 3,413 (13.1%) |
| **Urbanized municipalities** | 4,083 (25.0%) |  | 5,961 (22.9%) |
| **Rural areas** | 2,743 (16.8%) |  | 4,444 (17.0%) |
| **Green space 1000 m buffer** | 8.1% ± 11.9 | 25,691 | 8.1% ± 12.1 |

**^a^ n = data available for 14,899 participants**

**^b^ n = data available for 16,330 participants**

**Supplementary table 2: The mental health indicators with their scores and uses.**

| Questionnaire | Indicator | Score | Use |
| --- | --- | --- | --- |
| GHQ-12 | Indicator of mental well-being. | 12 questions  Answers: ‘better than usual’, ‘as good as usual’, ‘less than usual’, ‘much less than usual’  Ranking: [0 0 1 1]  The sum (from 0-12) represents the global GHQ-score. | 1. A cut-off point of 2 or more is used to identify those participants with at least 2 ‘abnormal’ psychological complaints, indicating a possible psychopathology.  2. A cut-off point of 4 or more is used to identify those participants with at least 4 ‘abnormal’ psychological complaints, indicating the more severe cases. |
| SF-36 | Indicator of the positive psychological health (vitality) | 4 questions  Answers: ‘always’, ‘most of the time’, ‘sometimes’, ‘rarely’, ‘never’  Ranking: [5 4 3 2 1] for the first two questions and [1 2 3 4 5] for the last two questions.  The average of the scores is converted to a scale from 0 to 100. | The average score + the standard deviation is used as the cut-off point to indicate those who have an vitality score well above average. |
| SCL-90-R | Indicator of depressive and generalized anxiety disorder in HIS 2008 and 2013 | 13 and 10 questions for depressive and generalized anxiety disorder respectively  Answers: ‘Not at all’, ‘a little bit’, ‘moderately’, ‘quite a bit’, ‘extremely’  Ranking: [0 1 2 3 4]  The sum of the questions is dived by the number of items in each scale. | A score higher than 1 is used to identify those participants with a depressive or generalized anxiety disorder. |

**GHQ-12, General health questionnaire; SF-36, Short form health survey; SCL-90-R: Symptom checklist – 90 – revised**

**Supplementary table 3: Results of the main and the sensitivity analyses.** Results are presented as the odds ratio (95% CI) of having a mental health condition or disorder or having a poor self-rated health for an IQR increase in air pollution. Multivariate logistic regressions were adjusted for age, gender, year, region, highest education of the household, household composition, country of birth, smoking status and the quadratic term of age when both the linear and quadratic term were significant. Sensitivity analyses were additionally adjusted for physical activity, urbanization, green space in a 1000 m buffer (separately).

|  | **Main model** | | **+ Physical activity** | | **+ Urbanisation** | | **+ Green space** | |
| --- | --- | --- | --- | --- | --- | --- | --- | --- |
|  | OR and 95% CI | *p*-value | OR and 95% CI | *p*-value | OR and 95% CI | *p*-value | OR and 95% CI | *p*-value |
| **Psychological distress** |  |  |  |  |  |  |  |  |
| **PM_2.5_** | 1.07 (0.98, 1.17) | 0.161 | 1.05 (0.95, 1.15) | 0.343 | 1.06 (0.95, 1.17) | 0.292 | 1.05 (0.96, 1.16) | 0.264 |
| **BC** | 1.02 (0.97, 1.08) | 0.400 | 1.02 (0.96, 1.08) | 0.511 | 1.02 (0.95, 1.08) | 0.650 | 1.02 (0.96, 1.08) | 0.532 |
| **NO_2_** | 1.06 (0.99, 1.14) | 0.076 | 1.05 (0.98, 1.13) | 0.199 | 1.07 (0.98, 1.16) | 0.146 | 1.06 (0.99, 1.13) | 0.120 |
| **Severe psychological distress** |  |  |  |  |  |  |  |  |
| **PM_2.5_** | 1.07 (0.96, 1.19) | 0.227 | 1.04 (0.93, 1.16) | 0.520 | 1.01 (0.89, 1.14) | 0.899 | 1.07 (0.96, 1.20) | 0.239 |
| **BC** | 0.99 (0.93, 1.05) | 0.733 | 0.98 (0.91, 1.05) | 0.512 | 0.95 (0.88, 1.02) | 0.145 | 0.99 (0.93, 1.05) | 0.694 |
| **NO_2_** | 1.04 (0.96, 1.13) | 0.336 | 1.02 (0.93, 1.11) | 0.736 | 0.98 (0.88, 1.08) | 0.639 | 1.04 (0.96, 1.13) | 0.354 |
| **Suicidal ideation in past 12 months** |  |  |  |  |  |  |  |  |
| **PM_2.5_** | 1.04 (0.86, 1.26) | 0.699 | 1.02 (0.83, 1.25) | 0.876 | 1.02 (0.82, 1.27) | 0.861 | 1.06 (0.87, 1.29) | 0.562 |
| **BC** | 1.00 (0.90, 1.11) | 0.974 | 0.96 (0.86, 1.07) | 0.452 | 0.99 (0.88, 1.12) | 0.915 | 1.01 (0.91, 1.12) | 0.872 |
| **NO_2_** | 1.03 (0.90, 1.18) | 0.678 | 0.99 (0.86, 1.15) | 0.912 | 1.01 (0.85, 1.21) | 0.884 | 1.04 (0.91, 1.20) | 0.568 |
| **Suboptimal vitality** |  |  |  |  |  |  |  |  |
| **PM_2.5_** | 1.27 (1.13, 1.42) | <0.0001 | 1.23 (1.08, 1.39) | 0.001 | 1.31 (1.14, 1.49) | <0.0001 | 1.27 (1.12, 1.43) | 0.0001 |
| **BC** | 1.07 (0.99, 1.15) | 0.102 | 1.05 (0.97, 1.13) | 0.263 | 1.05 (0.96, 1.15) | 0.257 | 1.06 (0.98, 1.15) | 0.129 |
| **NO_2_** | 1.13 (1.03, 1.23) | 0.008 | 1.10 (1.00, 1.21) | 0.055 | 1.16 (1.03, 1.31) | 0.014 | 1.12 (1.03, 1.23) | 0.012 |
| **Poor self-rated health** |  |  |  |  |  |  |  |  |
| **PM_2.5_** | 1.20 (1.09, 1.32) | 0.0003 | 1.15 (1.03, 1.28) | 0.010 | 1.21 (1.09, 1.36) | 0.0007 | 1.19 (1.08, 1.32) | 0.0006 |
| **BC** | 1.09 (1.03, 1.15) | 0.004 | 1.05 (0.99, 1.12) | 0.112 | 1.09 (1.02, 1.16) | 0.012 | 1.08 (1.02, 1.15) | 0.008 |
| **NO_2_** | 1.14 (1.06, 1.22) | 0.0006 | 1.08 (1.00, 1.17) | 0.052 | 1.17 (1.07, 1.29) | 0.001 | 1.13 (1.05, 1.22) | 0.001 |
| **Depressive disorder** |  |  |  |  |  |  |  |  |
| **PM_2.5_** | 1.19 (1.00, 1.41) | 0.045 | 1.14 (0.95, 1.37) | 0.157 | 1.25 (1.02, 1.54) | 0.029 | 1.20 (1.01, 1.43) | 0.044 |
| **BC** | 1.08 (1.00, 1.17) | 0.058 | 1.06 (0.98, 1.15) | 0.163 | 1.10 (1.00, 1.20) | 0.050 | 1.08 (1.00, 1.17) | 0.060 |
| **NO_2_** | 1.07 (0.96, 1.19) | 0.230 | 1.03 (0.92, 1.15) | 0.650 | 1.09 (0.95, 1.26) | 0.215 | 1.07 (0.96, 1.19) | 0.241 |
| **Generalized anxiety disorder** |  |  |  |  |  |  |  |  |
| **PM_2.5_** | 1.19 (0.97, 1.47) | 0.095 | 1.21 (0.97, 1.51) | 0.091 | 1.33 (1.03, 1.71) | 0.027 | 1.23 (0.99, 1.52) | 0.067 |
| **BC** | 1.09 (0.99, 1.20) | 0.095 | 1.08 (0.98, 1.19) | 0.117 | 1.11 (0.99, 1.25) | 0.068 | 1.09 (0.99, 1.20) | 0.076 |
| **NO_2_** | 1.06 (0.92, 1.21) | 0.417 | 1.05 (0.91, 1.20) | 0.530 | 1.12 (0.94, 1.32) | 0.205 | 1.06 (0.93, 1.22) | 0.373 |

**Supplementary table 4: The results of physical activity associated with mental and self-rated health and air pollution.** Results of the analysis mediator - outcome are presented as the odds ratio (95% CI) of having a mental health condition or disorder or having a poor self-rated health when in the sedentary group compared to the active group. Results of the analysis exposure - mediator are presented as the odds ratio (95% CI) of having a sedentary behavior when exposed to an IQR increase in air pollution. The multivariate logistic regressions were adjusted for age, gender, year, region, highest education of the household, household composition, country of birth, smoking status and the quadratic term of age when the result of the analysis showed that both the linear and quadratic term were significant.

| **Exposure / Outcome** | **OR and 95 % CI** | ***p*-value** |
| --- | --- | --- |
| **Psychological distress** | 1.63 (1.50, 1.77) | < 0.0001 |
| **Severe psychological distress** | 1.98 (1.79, 2.18) | < 0.0001 |
| **Suicidal ideation** | 1.88 (1.59, 2.23) | < 0.0001 |
| **Suboptimal vitality** | 2.22 (1.95, 2.52) | < 0.0001 |
| **Poor self-rated health** | 2.72 (2.48, 2.97) | < 0.0001 |
| **Depressive disorder** | 2.11 (1.83, 2.43) | < 0.0001 |
| **Generalized anxiety disorder** | 1.77 (1.49, 2.10) | < 0.0001 |
| **PM_2.5_**  **BC** | 1.29 (1.17, 1.42)  1.14 (1.07, 1.21) | < 0.0001  < 0.0001 |
| **NO_2_** | 1.23 (1.15, 1.33) | 0.0002 |

**Supplementary table 5: The effects of air pollution exposure on mental and self-rated health mediated through physical activity.**

|  | **NDE** | **95% CI** | ***p*-value** | **NIE** | **95% CI** | ***p*-value** | **TE** | **95% CI** | ***p*-value** | **% MED** | **95% CI** | ***p*-value** |
| --- | --- | --- | --- | --- | --- | --- | --- | --- | --- | --- | --- | --- |
| **PM_2.5_** |  |  |  |  |  |  |  |  |  |  |  |  |
| Psychological distress | 1.05 | 0.95, 1.14 | 0.326 | 1.03 | 1.02, 1.04 | <0.0001 | 1.07 | 0.98, 1.17 | 0.128 | 37.4 | -8.5, 83.3 | 0.110 |
| Severe psychological distress | 1.04 | 0.92, 1.15 | 0.515 | 1.04 | 1.02, 1.06 | <0.0001 | 1.08 | 0.96, 1.20 | 0.200 | 51.4 | -21.9, 124.8 | 0.169 |
| Suicidal ideation | 1.02 | 0.82, 1.22 | 0.877 | 1.04 | 1.02, 1.06 | <0.0001 | 1.05 | 0.85, 1.26 | 0.610 | 70.8 | -188.1, 329.6 | 0.592 |
| Suboptimal vitality | 1.22 | 1.07, 1.37 | 0.003 | 1.05 | 1.03, 1.07 | <0.0001 | 1.28 | 1.12, 1.44 | 0.0005 | 20.8 | 9.7, 31.8 | 0.0002 |
| Poor self-rated health | 1.14 | 1.03, 1.26 | 0.017 | 1.06 | 1.03, 1.08 | <0.0001 | 1.21 | 1.08, 1.33 | 0.001 | 31.1 | 13.5, 48.7 | 0.0005 |
| Depressive disorder | 1.14 | 0.95, 1.33 | 0.150 | 1.05 | 1.02, 1.07 | <0.0001 | 1.19 | 0.99, 1.39 | 0.059 | 27.3 | 1.5, 53.2 | 0.038 |
| Generalized anxiety disorder | 1.21 | 0.96, 1.46 | 0.093 | 1.03 | 1.01, 1.05 | 0.0006 | 1.25 | 0.99, 1.51 | 0.055 | 15.2 | 0.6, 29.8 | 0.042 |
| **BC** |  |  |  |  |  |  |  |  |  |  |  |  |
| Psychological distress | 1.02 | 0.96, 1.08 | 0.512 | 1.01 | 1.01, 1.02 | <0.0001 | 1.03 | 0.98, 1.09 | 0.253 | 43.8 | -29.4, 117.0 | 0.241 |
| Severe psychological distress | 0.98 | 0.91, 1.05 | 0.518 | 1.02 | 1.01, 1.03 | <0.0001 | 1.00 | 0.93, 1.07 | 0.941 | -754.5 | -20913.0, 19404.0 | 0.942 |
| Suicidal ideation | 0.95 | 0.84, 1.07 | 0.450 | 1.02 | 1.01, 1.03 | <0.0001 | 0.97 | 0.85, 1.10 | 0.670 | -73.7 | -425.4, 277.9 | 0.681 |
| Suboptimal vitality | 1.05 | 0.96, 1.13 | 0.282 | 1.03 | 1.01, 1.04 | <0.0001 | 1.07 | 0.98, 1.16 | 0.106 | 36.0 | -5.1, 77.1 | 0.086 |
| Poor self-rated health | 1.05 | 0.98, 1.11 | 0.159 | 1.03 | 1.02, 1.04 | <0.0001 | 1.08 | 1.01, 1.15 | 0.026 | 40.1 | 6.4, 73.7 | 0.020 |
| Depression disorder | 1.06 | 0.97, 1.15 | 0.199 | 1.02 | 1.01, 1.03 | 0.0004 | 1.08 | 0.99, 1.17 | 0.088 | 26.7 | -3.7, 57.1 | 0.085 |
| Generalized anxiety disorder | 1.08 | 0.97, 1.19 | 0.144 | 1.01 | 1.00, 1.02 | 0.003 | 1.10 | 0.99, 1.21 | 0.090 | 15.3 | -3.0, 33.6 | 0.102 |
| **NO2** |  |  |  |  |  |  |  |  |  |  |  |  |
| Psychological distress | 1.05 | 0.98, 1.12 | 0.195 | 1.02 | 1.01, 1.03 | <0.0001 | 1.07 | 1.00, 1.14 | 0.058 | 33.5 | 0.02, 66.9 | 0.050 |
| Severe psychological distress | 1.01 | 0.93, 1.10 | 0.733 | 1.03 | 1.02, 1.05 | <0.0001 | 1.05 | 0.96, 1.14 | 0.284 | 69.4 | -51.3, 190.1 | 0.260 |
| Suicidal ideation | 0.99 | 0.84, 1.13 | 0.876 | 1.03 | 1.02, 1.05 | <0.0001 | 1.02 | 0.87, 1.17 | 0.785 | 155.3 | -935.4, 1246.0 | 0.780 |
| Suboptimal vitality | 1.10 | 0.99, 1.21 | 0.069 | 1.04 | 1.02, 1.06 | <0.0001 | 1.14 | 1.03, 1.26 | 0.014 | 30.9 | 9.1, 52.7 | 0.006 |
| Poor self-rated health | 1.08 | 0.99, 1.16 | 0.069 | 1.05 | 1.03, 1.07 | <0.0001 | 1.13 | 1.04, 1.22 | 0.005 | 40.1 | 14.6, 65.6 | 0.002 |
| Depression disorder | 1.03 | 0.91, 1.14 | 0.651 | 1.03 | 1.02, 1.05 | <0.0001 | 1.06 | 0.94, 1.18 | 0.325 | 55.7 | -49.5, 160.8 | 0.299 |
| Generalized anxiety disorder | 1.05 | 0.90, 1.19 | 0.523 | 1.02 | 1.01, 1.04 | 0.0007 | 1.07 | 0.92, 1.22 | 0.350 | 33.2 | -33.6, 100.0 | 0.330 |

The table shows the odds ratios (95% CI) of the natural direct effect (NDE), the natural indirect effect (NIE), the total effect (TE), and the proportion of mediation (% Med). The mediation model was adjusted for age, gender, year of participation, region, country of birth, household composition, smoking status and highest educational level of the household.

**Supplementary table 6: Association between air pollution and depressive and generalized anxiety disorder where the outcomes are classified as tertiles.** Class 0 indicates the lowest scores, class 2 indicates the highest scores. Results of the multivariate logistic regressions are presented as the odds ratio (95% CI) of being in class 1 or 2 compared with 0 (reference category) for an IQR increment in air pollution exposure. Analysis were adjusted for gender, year, region, highest education of the household, household composition, country of birth, and smoking status.

|  | **Class** | **OR and 95% CI** | ***p*-value** |
| --- | --- | --- | --- |
| **Depressive disorder** |  |  |  |
| **PM_2.5_** | 1 vs 0  2 vs 0 | 1.15 (1.01, 1.30)  1.15 (1.00, 1.32) | 0.034  0.051 |
| **BC** | 1 vs 0  2 vs 0 | - 1. (0.95, 1.08)   0.97 (0.91, 1.05) | 0.692  0.455 |
| **NO_2_** | 1 vs 0  2 vs 0 | 1.04 (0.95, 1.13)  1.01 (0.91, 1.11) | 0.436  0.912 |
| **Generalize anxiety disorder** |  |  |  |
| **PM_2.5_** | 1 vs 0  2 vs 0 | 1.16 (0.80, 1.67)  1.37 (0.95, 1.95) | 0.442  0.089 |
| **BC** | 1 vs 0  2 vs 0 | 0.98 (0.91, 1.05)  1.00 (0.94, 1.07) | 0.504  0.983 |
| **NO_2_** | 1 vs 0  2 vs 0 | 0.99 (0.91, 1.08)  0.99 (0.91, 1.08) | 0.865  0.827 |

**Supplementary table 7: Results of the effect modification of age.** Stratification was done when the interaction-term was found significant (*p for interaction* ≤ 0.10). Results of the multivariate logistic regressions are presented as the odds ratio (95% CI) of having a mental health condition or disorder or a poor self-rated health for an IQR increment in air pollution exposure. Analysis were adjusted for gender, year, region, highest education of the household, household composition, country of birth, and smoking status.

|  | **P_int_ Age** | **Age 15-29**  **n = 2,548** | **Age 30-44**  **n = 4,005** | **Age 45-59**  **n = 4,586** | **Age +60**  **n = 5,316** |
| --- | --- | --- | --- | --- | --- |
|  |  | **OR and 95% CI**  ***p*-value** | **OR and 95% CI**  ***p*-value** | **OR and 95% CI**  ***p*-value** | **OR and 95% CI**  ***p*-value** |
| **Psychological distress** |  |  |  |  |  |
| **PM_2.5_** | 0.0001 | 0.96 (0.77, 0.19)  *p* = 0.713 | 0.94 (0.79, 0.13)  *p* = 0.518 | 0.12 (0.95, 1.32)  *p* = 0.186 | 1.15 (0.98, 1.36)  *p* = 0.086 |
| **BC** | 0.005 | 1.01 (0.88, 1.15)  *p* = 0.943 | 0.97 (0.88, 1.07)  *p* = 0.566 | 1.07 (0.96, 1.20)  *p* = 0.244 | 1.04 (0.94, 1.15)  *p* = 0.435 |
| **NO_2_** | 0.077 | 1.02 (0.87, 1.20)  *p* = 0.812 | 1.00 (0.88, 1.14)  *p* = 0.986 | 1.12 (0.98, 1.27)  *p* = 0.092 | 1.06 (0.94, 1.19)  *p* = 0.355 |
| **Severe psychological distress** |  |  |  |  |  |
| **PM_2.5_** | 0.0001 | 0.97 (0.75, 1.26)  *p* = 0.817 | 0.95 (0.76, 1.18)  *p* = 0.628 | 1.04 (0.86, 1.28)  *p* = 0.674 | 1.24 (1.02, 1.51)  *p* = 0.028 |
| **BC** | 0.002 | 0.88 (0.75, 1.05)  *p* = 0.159 | 0.97 (0.87, 1.09)  *p* = 0.643 | 0.99 (0.86, 1.14)  *p* = 0.888 | 1.05 (0.93, 1.19)  *p* = 0.429 |
| **NO_2_** | 0.044 | 0.98 (0.80, 1.20)  *p* = 0.837 | 1.00 (0.85, 1.16)  *p* = 0.955 | 1.04 (0.89, 1.22)  *p* = 0.620 | 1.08 (0.94, 1.25)  *p* = 0.280 |
| **Suicidal ideation in past 12 months** |  |  |  |  |  |
| **PM_2.5_** | 0.125 | / | / | / | / |
| **BC** | 0.071 | 0.83 (0.62, 1.11)  *p* = 0.215 | 0.97 (0.80, 1.17)  *p* = 0.726 | 1.14 (0.92, 1.42)  *p* = 0.232 | 1.11 (0.90, 1.37)  *p* = 0.338 |
| **NO_2_** | 0.016 | 0.83 (0.60, 1.15)  *p* = 0.261 | 1.03 (0.78, 1.35)  *p* = 0.851 | 1.11 (0.86, 1.44)  *p* = 0.420 | 1.20 (0.92, 1.57)  *p* = 0.178 |
| **Suboptimal vitality** |  |  |  |  |  |
| **PM_2.5_** | 0.298 | / | / | / | / |
| **BC** | 0.182 | / | / | / | / |
| **NO_2_** | 0.668 | / | / | / | / |
| **Poor self-rated health** |  |  |  |  |  |
| **PM_2.5_** | 0.031 | 1.20 (0.82 – 1.76)  *p* = 0.357 | 0.91 (0.73 – 1.14)  *p* = 0.422 | 1.17 (0.98 – 1.39)  *p* = 0.090 | 1.36 (1.18 – 1.57)  *p* < 0.0001 |
| **BC** | 0.073 | 1.18 (1.00 – 1.40)  *p* = 0.047 | 0.93 (0.83 – 1.04)  *p* = 0.210 | 1.03 (0.92 – 1.16)  *p* = 0.603 | 1.19 (1.08 – 1.31)  *p* = 0.0003 |
| **NO_2_** | 0.366 | / | / | / | / |
| **Depressive disorder** |  |  |  |  |  |
| **PM_2.5_** | 0.183 | / | / | / | / |
| **BC** | 0.212 | / | / | / | / |
| **NO_2_** | 0.254 | / | / | / | / |
| **Generalized anxiety disorder** |  |  |  |  |  |
| **PM_2.5_** | 0.512 | / | / | / | / |
| **BC** | 0.805 | / | / | / | / |
| **NO_2_** | 0.858 | / | / | / | / |

**Supplementary table 8: Results of the effect modification of sex.** Stratification was done when the interaction-term was found significant (*p for interaction* ≤ 0.10). Results of the multivariate logistic regressions are presented as the odds ratio (95% CI) of having a mental health condition or disorder or a poor self-rated health for an IQR increment in air pollution exposure. Analysis were adjusted for age, year, region, highest education of the household, household composition, country of birth, smoking status, and the quadratic term of age when both the linear and quadratic term were significant.

|  | **P_int_ Age** | **Men**  **n = 7,813** | **Women**  **n = 8,642** |
| --- | --- | --- | --- |
|  |  | **OR and 95% CI**  ***p*-value** | **OR and 95% CI**  ***p*-value** |
| **Psychological distress** |  |  |  |
| **PM_2.5_** | 0.056 | 1.12 (0.98, 1.28)  *p* = 0.087 | 1.03 (0.92, 0.15)  *p* = 0.663 |
| **BC** | 0.047 | 1.04 (0.97, 1.13)  *p* = 282 | 1.01 (0.94, 1.08)  *p* = 877 |
| **NO_2_** | 0.025 | 1.10 (1.00, 1.21)  *p* = 0.053 | 1.03 (0.95, 1.13)  *p* = 0.448 |
| **Severe psychological distress** |  |  |  |
| **PM_2.5_** | 0.437 | / | / |
| **BC** | 0.223 | / | / |
| **NO_2_** | 0.076 | 1.08 (0.96, 1.22)  *p* = 0.189 | 1.01 (0.91, 1.12)  *p* = 0.849 |
| **Suicidal ideation in past 12 months** |  |  |  |
| **PM_2.5_** | 0.527 | / | / |
| **BC** | 0.440 | / | / |
| **NO_2_** | 0.528 | / | / |
| **Suboptimal vitality** |  |  |  |
| **PM_2.5_** | 0.115 | / | / |
| **BC** | 0.194 | / | / |
| **NO_2_** | 0.103 | / | / |
| **Poor self-rated health** |  |  |  |
| **PM_2.5_** | 0.236 | / | / |
| **BC** | 0.389 | / | / |
| **NO_2_** | 0.271 | / | / |
| **Depressive disorder** |  |  |  |
| **PM_2.5_** | 0.049 | 1.00 (0.77 – 1.32)  *p* = 0.976 | 1.30 (1.06 – 1.59)  *p* = 0.012 |
| **BC** | 0.173 | / | / |
| **NO_2_** | 0.256 | / | / |
| **Generalized anxiety disorder** |  |  |  |
| **PM_2.5_** | 0.092 | 1.04 (0.73, 1.48)  *p* = 0.820 | 1.27 (1.00, 1.62)  *p* = 0.055 |
| **BC** | 0.619 | / | / |
| **NO_2_** | 0.536 | / | / |

**Supplementary table 9: Results of the effect modification of socio-economic status (SES).** Stratification was done when the interaction-term was found significant (*p for interaction* ≤ 0.10). Results of the multivariate logistic regressions are presented as the odds ratio (95% CI) of having a mental health condition or disorder or a poor self-rated health for an IQR increment in air pollution exposure. Analysis were adjusted for age, gender, year, region, highest education of the household, household composition, country of birth, smoking status and the quadratic term of age when both the linear and quadratic term were significant.

|  | **P_int_ SES** | **Up to lower secondary school**  **n = 3,543** | **Higher secondary school**  **n = 5,149** | **College or university**  **n = 7,763** |
| --- | --- | --- | --- | --- |
|  |  | **OR and 95% CI**  ***p*-value** | **OR and 95% CI**  ***p*-value** | **OR and 95% CI**  ***p*-value** |
| **Psychological distress** |  |  |  |  |
| **PM_2.5_** | 0.130 | / | / | / |
| **BC** | 0.080 | 1.02 (0.91, 1.14)  *p* = 0.724 | 1.05 (0.96, 1.16)  *p* = 0.275 | 0.99 (0.91, 1.08)  *p* = 0.894 |
| **NO_2_** | 0.105 | / | / | / |
| **Severe psychological distress** |  |  |  |  |
| **PM_2.5_** | 0.733 | / | / | / |
| **BC** | 0.917 | / | / | / |
| **NO_2_** | 0.854 | / | / | / |
| **Suicidal ideation in past 12 months** |  |  |  |  |
| **PM_2.5_** | 0.666 | / | / | / |
| **BC** | 0.615 | / | / | / |
| **NO_2_** | 0.575 | / | / | / |
| **Suboptimal vitality** |  |  |  |  |
| **PM_2.5_** | 0.984 | / | / | / |
| **BC** | 0.772 | / | / | / |
| **NO_2_** | 0.540 | / | / | / |
| **Poor self-rated health** |  |  |  |  |
| **PM_2.5_** | 0.143 | / | / | / |
| **BC** | 0.282 | / | / | / |
| **NO_2_** | 0.695 | / | / | / |
| **Depressive disorder** |  |  |  |  |
| **PM_2.5_** | 0.982 | / | / | / |
| **BC** | 0.985 | / | / | / |
| **NO_2_** | 0.919 | / | / | / |
| **Generalized anxiety disorder** |  |  |  |  |
| **PM_2.5_** | 0.947 | / | / | / |
| **BC** | 0.411 | / | / | / |
| **NO_2_** | 0.587 | / | / | / |

**Supplementary table 10: Overview of all literature presented in the discussion.**

| **Outcome** | **Pollutants + concentration (Mean ± SD)** | **Location** | **Subjects** | **Health effect** | **Reference** |
| --- | --- | --- | --- | --- | --- |
| Psychological distress | Annual  PM_2.5_ (11.35 µg/m³ ± 2.93)  PM_10_ (NA)  NO_2_ (NA) | U.S. | 15+ adolescents and adults | Higher PM_2.5_ exposure is associated with higher odds of psychological distress. No association found for PM_10_ and NO_2_. | [1] |
|  | Long-term ^a^  PM_2.5_, (16.7 µg/m³ (0.83))  PM_10_ (24.4 µg/m³ (1.24))  NO_2_ (23.4 µg/m³ (7.85)) | The Netherlands | Adults | Higher exposure concentrations were associated with higher odds of psychological distress. | [2] |
| Suicide rate/ ideation/attempt | Short-term  PM_10_ (29.0 µg/m³ ± 15.20)  O_3_ (61.8 µg/m³ ± 30.30) | Belgium | Children and adults | During summer, people were more likely to commit suicide when PM_10_ and ozone concentrations were high. | [3] |
|  | Short-term ^b^  O_3_ (24.61 ppb (12.77)) | Taiwan | Children and adults | The risk of committing a suicide attempt increased with higher O_3_ levels. | [4] |
|  | Short-term  PM_2.5_ (25.06 µg/m³ ± 10.63)  PM_10_ (50.47 µg/m³ ± 20.43)  O_3_ (81.39 ppb ± 28.04)  NO_2_ (27.75 ppb ± 8.53) | Mexico | Children and adults | No significant association was found. | [5] |
|  | Long-term ^b^  PM_10_ (55.11 µg/m³ (7.48))  NO_2_ (25.64 ppb (11.78)) | Korea | Adults | Risk of suicide was higher in the highest (PM_10_) and third (NO_2_) quartile. | [6] |
|  | Annual  PM_10_ (48.6 µg/m³ ± 8.30)  NO_2_ (24.0 ± 7.80) | Korea | Adults | Higher odds of suicidal ideation in the highest quartile of exposure. | [7] |
|  | Short-term  PM_10_ (51.63 µg/m³ ± 18.91)  O_3_ (0.024 ppm ± 0.008)  NO_2_ (0.022 ppm ± 0.005) | South-Korea | Adults and children | PM_10_ and O_3_ exposure were associated with higher suicide rates at time lags 0 and 4. No association was found for NO_2_. | [8] |
|  | Monthly ^b^  PM_10_ (49.5 µg/m³ (15.00))  O_3_ (19.4 ppb (7.80)) | Taiwan | Adolescents and adults | PM_10_ and O_3_ exposure were associated with a higher suicide rate. | [9] |
| Vitality | Monthly  PM (22.5 µg/m³ ± 8.00)  NOx (45.0 ppb ± 21.30)  O_x_ (21.0 ± 5.70) | Japan | Adults | Higher exposure to O_x_ was associated with a lower vitality score. For PM_2,5_ there was only an association in the crude analysis and no association was found for NO_x_. | [10] |
| **Supplementary table 10 continued.** | |  |  |  |  |
| **Outcome** | **Pollutants + concentration (Mean ± SD)** | **Location** | **Subjects** | **Health effect** | **Reference** |
| Depressive disorder ^a^  and Anxiety disorder | Annual  PM_2.5_ (16.8 (2.70)  NO_2_ (57.3 (12.00)  PM_10_ (37.7 (5.00) | Spain | Adults (85% had at least one parent diagnosed with dementia) | Higher exposure was associated with increased odds of depressive disorder.  No association was found between air pollution and anxiety | [11] |
| Depression score | Long-term  PM_10_ (43.7 µg/m³ ± 23.7)  NO_2_ (36.2 ppb ± 12.1)  O_3_ (48.1 ppb ± 27) | Korea | Elderly | Higher 3-day moving average concentrations were associated with increased odds of depressive disorder. | [12] |
| Anxiety disorder | Annual  PM_2.5_ (11.1 µg/m³ ± 3.0 and  8.8 µg/m³ ± 2.3 in the two waves) | U.S. | Adults (57–85 years) | An increase in PM_2.5_ was significantly associated with anxiety symptoms, with the largest increase for 180-days moving average | [13] |
|  | Monthly  PM_2.5_ (12.74 µg/m³ ± 4.18) | U.S. | Adult women | Exposure to PM_2.5_ was associated with high symptoms of anxiety | [14] |
| Self-rated health | Monthly  NO_2_ (NA)  PM_2.5_ (NA) | Japan | Adults (40 – 79 years) | Higher exposure to NO_2_ and PM was associated with a decreased overall health score but no association was found between PM_2.5_ and overall health. | [15] |

**PM = Particulate matter**

**^a^ Concentrations presented as median (IQR)**

**^b^ Concentrations presented as mean (IQR)**

**
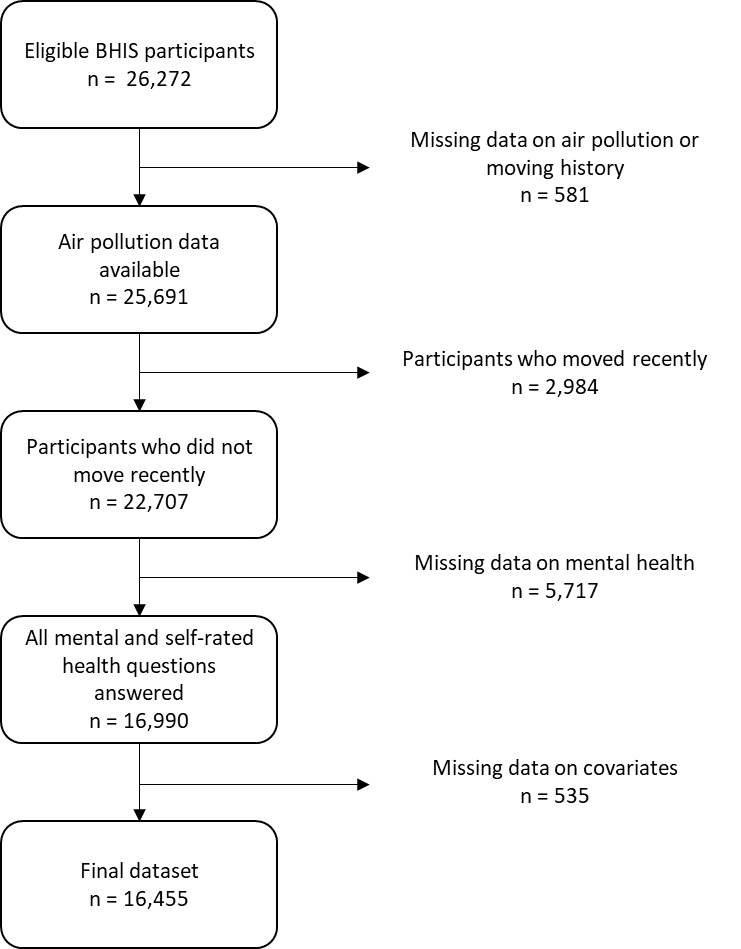
**

**Supplementary figure 1: Exclusion criteria.** The present study sample consisted of 16,463 BHIS participants. We excluded 9,799 participants because (1) data on air pollution and moving history was not available (n = 581); (2) persons living less than 1 year on the current address were excluded (n = 2,984); (3) participants who did not answer all mental or self-rated health questions and completed all information used as covariates in this study were excluded (n = 5,717 and 535, respectively).

**
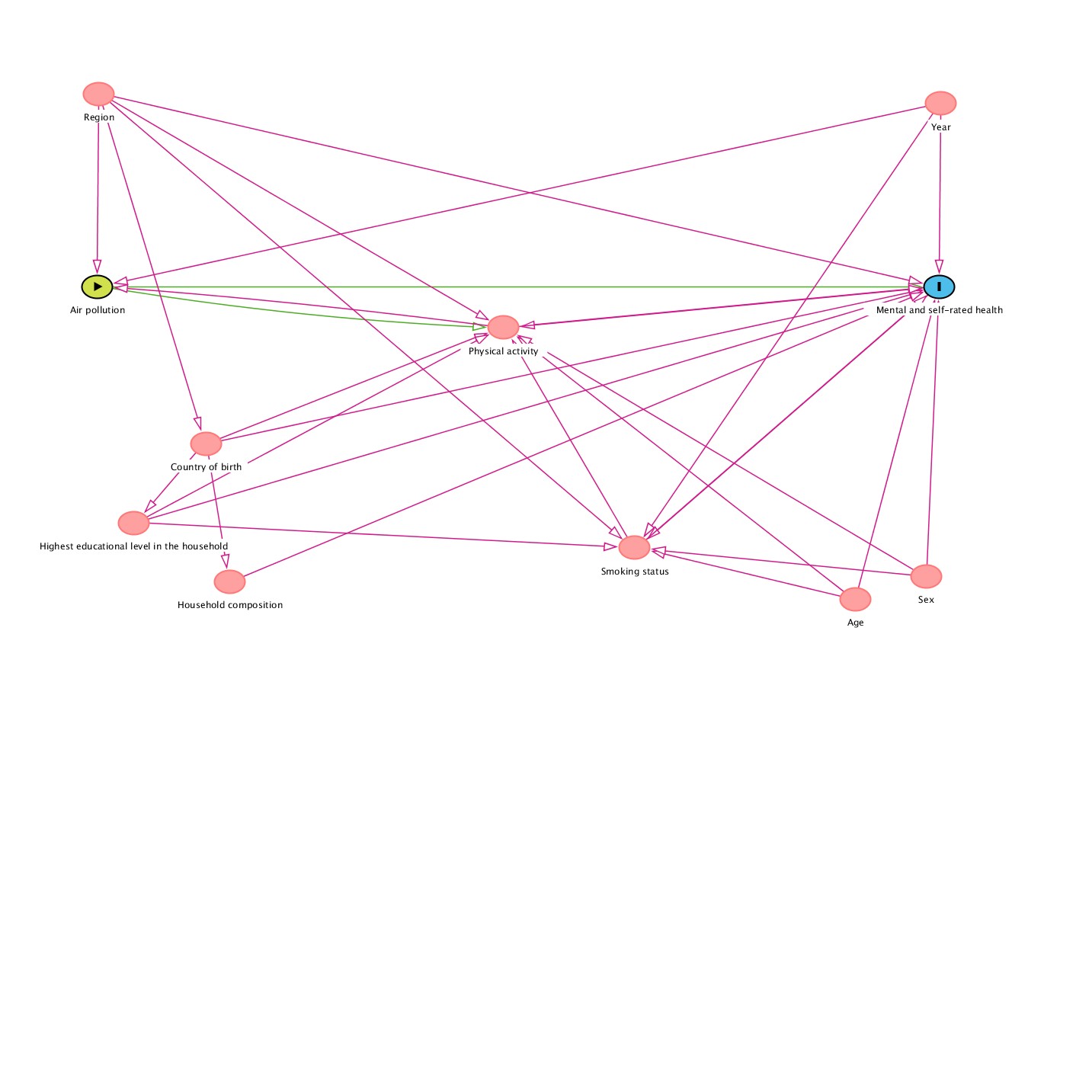
**

**Supplementary figure 2:** Directed acyclic graph (DAG) for the association between air pollution and mental and self-rated health. Red: Ancestor of exposure and outcome; Green line: causal path; Purple line: biasing path.

References

1. Sass V, Kravitz-Wirtz N, Karceski S, Hajat A, Crowder K, Takeuchi D. The Effects of Air Pollution on Individual Psychological Distress. Health Place. 2017;48:72-9. <https://doi.org/10.1016/j.healthplace.2017.09.006>.

2. Klompmaker JO, Hoek G, Bloemsma LD, Wijga AH, van den Brink C, Brunekreef B, et al. Associations of combined exposures to surrounding green, air pollution and traffic noise on mental health. Environment International. 2019;129:525-37. <https://doi.org/10.1016/j.envint.2019.05.040>.

3. Casas L, Cox B, Bauwelinck M, Nemery B, Deboosere P, Nawrot TS. Does air pollution trigger suicide? A case-crossover analysis of suicide deaths over the life span. European Journal of Epidemiology. 2017;32(11):973-81. <https://doi.org/10.1007/s10654-017-0273-8>.

4. Yang C-Y, Huang Y-T, Chiu H-F. Does ambient ozone air pollution trigger suicide attempts? A case cross-over analysis in Taipei. Journal of Toxicology and Environmental Health, Part A. 2019;82(10):638-44. <https://doi.org/10.1080/15287394.2019.1640980>.

5. Astudillo-García CI, Rodríguez-Villamizar LA, Cortez-Lugo M, Fernández-Niño JA. Air pollution and suicide in Mexico City: a time series analysis, 2000–2016. International Journal of Environmental Research and Public Health. 2019;16(16):2971. <https://doi.org/10.3390/ijerph16162971>.

6. Min J-y, Kim H-J, Min K-b. Long-term exposure to air pollution and the risk of suicide death: A population-based cohort study. Science of the Total Environment. 2018;628-629:573-9. <https://doi.org/10.1016/j.scitotenv.2018.02.011>.

7. Shin J, Park JY, Choi J. Long-term exposure to ambient air pollutants and mental health status: A nationwide population-based cross-sectional study. PloS One. 2018;13(4):e0195607. <https://doi.org/10.1371/journal.pone.0195607>.

8. Kim Y, Myung W, Won H-H, Shim S, Jeon HJ, Choi J, et al. Association between air pollution and suicide in South Korea: a nationwide study. PloS one. 2015;10(2):e0117929. <https://doi.org/10.1371/journal.pone.0117929>.

9. Yang AC, Tsai S-J, Huang NE. Decomposing the association of completed suicide with air pollution, weather, and unemployment data at different time scales. Journal of affective disorders. 2011;129(1-3):275-81. <https://doi.org/10.1016/j.jad.2010.08.010>.

10. Yamazaki S, Nitta H, Fukuhara S. Associations between exposure to ambient photochemical oxidants and the vitality or mental health domain of the health related quality of life. Journal of Epidemiology & Community Health. 2006;60(2):173-9. <https://doi.org/10.1136/jech.2005.039560>.

11. Vert C, Sánchez-Benavides G, Martínez D, Gotsens X, Gramunt N, Cirach M, et al. Effect of long-term exposure to air pollution on anxiety and depression in adults: A cross-sectional study. International Journal of Hygiene and Environmental Health. 2017;220(6):1074-80. <https://doi.org/10.1016/j.ijheh.2017.06.009>.

12. Lim Y-H, Kim H, Kim JH, Bae S, Park HY, Hong Y-C. Air pollution and symptoms of depression in elderly adults. Environmental Health Perspectives. 2012;120(7):1023-8. <https://doi.org/10.1289/ehp.1104100>.

13. Pun VC, Manjourides J, Suh H. Association of ambient air pollution with depressive and anxiety symptoms in older adults: results from the NSHAP study. Environmental Health Perspectives. 2017;125(3):342-8. <https://doi.org/10.1289/EHP494>.

14. Power MC, Kioumourtzoglou MA, Hart JE, Okereke OI, Laden F, Weisskopf MG. The relation between past exposure to fine particulate air pollution and prevalent anxiety: observational cohort study. BMJ. 2015;350:h1111. <https://doi.org/10.1136/bmj.h1111>

15. Nakao M, Yamauchi K, Mitsuma S, Omori H, Ishihara Y. Relationships between perceived health status and ambient air quality parameters in healthy Japanese: a panel study. BMC Public Health. 2019;19(1):620. <https://doi.org/10.1186/s12889-019-6934-7>.
